# Chemical compositions and biological activities of the oils from the genus *Taxus* and factors limiting the regeneration of endangered yews: a review

**DOI:** 10.55730/1300-0527.3480

**Published:** 2022-07-26

**Authors:** Sagar DHAKAL, Prem Kumar KHOSLA, Tokuma GETAHUN

**Affiliations:** 1 School of Biological and Environmental Sciences, Faculty of Sciences, Shoolini University, Solan (HP), India; 2 Department of Chemistry, Asella College of Teachers Education, Asella, Oromia, Ethiopia

**Keywords:** Medicinal plants, *Taxus* species, taxol and its precursors, endangered yews, essential oils, fatty acids, biological activities, regeneration factors

## Abstract

The genus *Taxus* (yews) is the largest genus of the family Taxaceae. It comprises about 24 species with 55 varieties distributed mainly in Asia, Europe, North Africa, and North America. In addition to the taxane diterpenoids and the cancer drug taxol, its species contain many essential oils with actual or potential biological activity. This review covers the chemical constituents as well as biological activities of these oils that have been studied in fourteen countries over 46 years (1975–2021). It also discusses the biotic and abiotic factors that limit the regeneration of these economically and medicinally important plants.

## 1. Introduction

The genus *Taxus* is the most important member of the family Taxaceae from a phytochemical perspective. Its species are in high demand for the extraction of taxol or related taxanes, a drug for the treatment of various cancers. Essential oils extracted from the studied *Taxus* plant parts were found to be composed mainly of alcohols. 1-Octen-3-ol, cis-3-hexen-1-ol, caryophyllene oxide, myrtenol, elemicin, trans-2-hexenal, α-pinene, and laminitol were the most frequent components with high concentrations of these essential oils [1–8]. Palmitic, oleic, linoleic, taxoleic, and α-linolenic acids were the most predominant and frequently reported fatty acid constituents of the oils (lipids) of *Taxus* plants from different regions [9–15]. The oils (essential oils and/or lipids) of the investigated plants of the genus *Taxus* have demonstrated powerful antifungal, antibacterial, antioxidant, and antihypertensive activities. However, the species of the genus *Taxus* are the most threatened and endangered plants in their geographical ranges [16,17]. Various factors are affecting the survival of these precious species and due to these, their regeneration was very poor. Therefore, to protect these plants, urgent conservation actions must be taken for all of the plants in their geographical sites. At the present time, the chemical constituents of the oils of only eight and the biological activities of the oils of only four *Taxus* species have been reported, which have been discussed in the later parts of this review.

**Figure 1:**
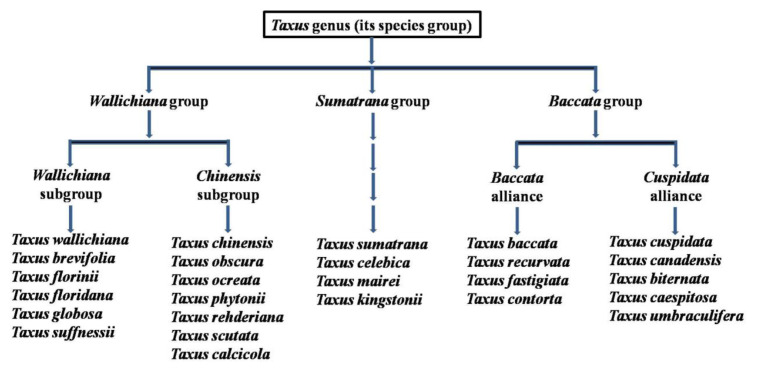
Classification of the genus *Taxus* [30,31].

**Figure 2:**
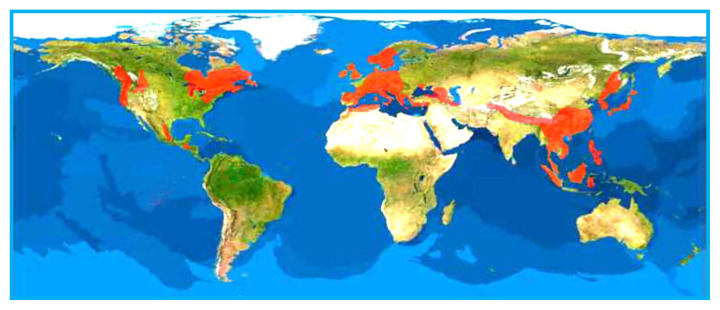
Worldwide distributions of the *Taxus* species.

*Taxus* (yews) is the largest genus of slow-growing long lived evergreen coniferous trees in the family Taxaceae. It comprises about 24 species with 55 varieties [18], distributed mainly in Asia (Pakistan, North India, Japan and China), Europe, North Africa and North America (see Figures 1 and 2) [19,20]. These plants are classified into three groups that are *Wallichiana*, *Baccata*, and *Sumatrana* (Figure 1) based on morphology and geographic distribution, such as European yews (Europe), Canadian yews (North America), and Himalayan yews (Asia) [21]. In Asia, Himalayan yews have a wide distribution in Hindu-Kush Himalaya (HKH) and neighboring regions, ranging from Afghanistan to Philippines [21]. Almost ten plants of the genus *Taxus* are distributed in this HKH region. These are *T. contorta* Griff., *T. contorta* Griff. var. *contorta*, *T. wallichiana* Zucc., *T. yunnanensis*, *T. mairei* (Lemée&H. Léveillé) S.Y. Hu ex T.S. Liu, *T. contorta* Griff. var. *mucronata* Spjut, *T. sumatrana* (Miq.) de Laubenfels, *T. phytonii* Spjut, *T. celebica* (Warb.) H.L. Li and *T. baccata* L. [22–24]. In North America, four *Taxus* species namely, *T. canadensis*, *T. floridana* (*T. globosa* var. *floridana sensu* Spjutis), *T. brevifolia*, and *T. globosa* Schltdl. are widely recognized [25]. In China, there are four species of the genus *Taxus* and one subspecies commonly found in the south-western and north-eastern regions of the country [26,27]. These are *T. yunnanensis* Cheng et L.K.Fu, *T. wallichiana* Zucc., *T. chinensis* (Pilg) Rehd., *T. chinensis var. mairei* (Lemee et Levl.) Cheng et L.K.Fu, and *T. cuspidata* Sieb.et Zucc. [27]. However, ten *Taxus* species such as *T. wallichiana* Zucc., *T. chinensis* (Pilg.) Rehder, *T. celebica* (Warb.) H.L. Li, *T. biternata* Spjut, *T. contorta* Griff., *T. mairei* (Lemée&Lév) S.Y.Huex T.S. Liu, *T. umbraculifera* (Sieb. ex Endl.) C. Lawson, *T. kingstonii* Spjut, *T. sumatrana* (Miq.) de Laub., *T. yunnanensis* W.C. Cheng & L.K. Fu, all of which are referred to as Chinese yews are reported to be native species [28]. Only one species, *Taxus baccata* L. (European yew) is found growing in Turkey [29].

Among all the identified *Taxus* species and subspecies or varieties, *T. contorta* Griff. (syn. *T. fuana*), *T. yunnanensis*, *T. baccata* subsp. *wallichiana*, *T. globosa* Schltdl., *T. cuspidata* Sieb.et Zucc., *T. chinensis* var. *mairei*, *T. wallichiana* var. *maireii*, *T. calcicola* L.M. Gao & Mich. Möller, *T. floridana* Nutt. ex Chapm., *T. florinii* Spjut, *T. chinensis* (Pilg.) Rehd. and *T. wallichiana* Zucc. are endangered/critically endangered species due to their low growth, regeneration, and overharvesting for several applications and medicinal uses [24,25,27,32–37]. These endangered species are also listed in https://threatenedconifers.rbge.org.uk/taxonomy/taxaceae/taxus.

**Figure 3:**
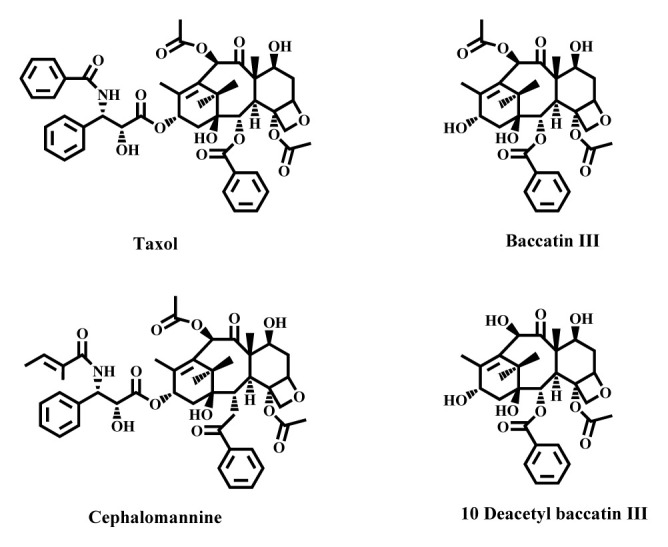
Chemical structure of taxol and its precursors encountered in *Taxus* species.

The leaves, roots, twigs, and dried bark of plants of the genus *Taxus* are used to relieve edema and remove toxicity from the body in traditional Chinese medicine (TCM) for a long time [26]. The leaves of *Taxus* plants have various types of medicinal uses to treat diseases like lung disorders, epilepsy, nervousness, hysteria, malaria, nephropathy, and diabetic nephropathy [19,38]. Various species of this genus have also been reported to exhibit a number of biological activities including antileukemic, analgesic, cytotoxic, antiinflammatory, sedative, anticancer, anticonvulsant, antipyretic, antibacterial, antimitotic, tranquilising, antifungal, and antiseptic [19,39]. Yews have also several applications in making of local beverages using their leaves extract, high-priced furniture, oil extraction, timber, fuel, traditional tea, and for woodcarving [34,36]. However, they gained global notoriety for their FDA (US) approved anticancer/cardiovascular drug paclitaxel (taxol) (Figure 3) which was recognized as one of the most effective and powerful antitumor agents [40]. Nowadays, as an option, this drug is largely produced from its precursors like 10-deacetyl baccatin III (10 DAB III), cephalomannine and baccatin III which are also more readily available in different parts of plants of the genus *Taxus* (see Table 1 and Figure 3) [41].

According to the literature survey, over 550 taxanes including taxol and a number of other different classes of compounds (e.g., phenolic compounds, abietanes, lignans, phytosterols, glycosides, fatty alcohol, steroids, flavonoids, sesquiterpene, and ecdysteroids) were isolated and reported from organic solvent extracts of different parts such as bark, needles, stems, leaves, seeds, twigs, heartwood, roots, and branches of various *Taxus* species (yews). Several reviews have also compiled these *Taxus* phytoconstituents [19,43–47]. However, only few *Taxus* plants have been studied concerning the chemical compositions and biological activities of their oils. To the best of our knowledge, there is no review paper published on these oils and their biological activities. Therefore, this review paper compiles a brief overview on the chemical compositions of the oils of *Taxus* plants and their biological activities reported in the published literature, using Google Scholar, Google, PubMed, and ScienceDirect databases which might be important in the pharmaceutical industries and drug formulation principles. Moreover, the review presents biotic and abiotic factors that limit the regeneration of these economically and medicinally important plants because many of them are listed as highly endangered species. Thus, the review is very useful for the researchers who have interest in performing further studies on *Taxus* plants.

### 1.1. Chemical constituents of oils of Taxus plants

The oils obtained from plants and their constituents are extensively used in cosmetics, detergents, perfumes, agriculture, soaps, foods, and pharmaceutical and other industries [66–71]. They are reported to have analgesic, antitumorigenic, repellent, insecticidal, AChE inhibitory, antifungal, antihypertensive, anticarcinogenic, antiviral, antiinflammatory, antibacterial, antioxidant, and antiparasitic properties [66,67,69]. Nowadays, the investigation on these oils and their constituents has been an interesting, attractive, and hot research area. Therefore, the analysis of the oils and their components including fatty acids is very important for complement of new information on plant applications, for the description of fresh perspective on the potential uses of these organic natural ingredients, and to help meet the requirements of the steadily increasing global edible oil markets.

**Figure 4:**
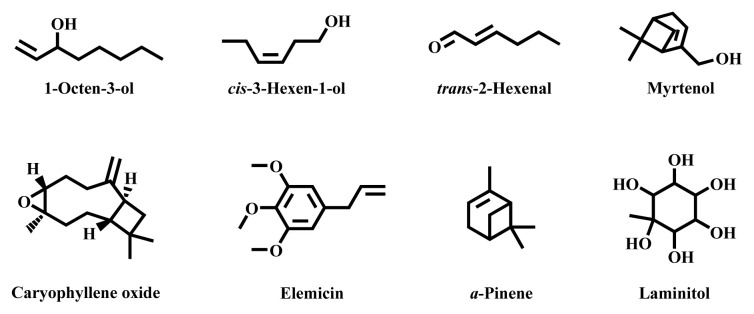
Chemical structures of most frequently reported constituents of essential oils from *Taxus* species.

The main target of this review is also to give an overview on the chemical constituents of the oils from different members of the genus *Taxus* worldwide. Table 2 shows the collection of the available literature data regarding the oil composition of these plants. According to the literature, among the identified *Taxus* plants, only eight of them, namely *T. chinensis*, *T. media*, *T. baccata*, *T. canadensis*, *T. chinensis* var. *mairei*, *T. cuspidata*, *T. wallichiana*, and *T. wallichiana* var *mairei* were investigated concerning the chemical constituent of their oils. Of these, *T. baccata* was the most studied plant. As presented in the table, the plant part, the most abundant components, country of study, and extraction and analysis methods of oils relating to different plants of this genus have been pointed out. Generally, the dominant chemical class of compounds of the essential oils (EOs) obtained by different methods from the species in the genus *Taxus* is alcohols (Table 2 and Figure 4) [1–5,72]. Alkanes, alkenes, aldehydes, ketones, flavonoids, fatty alcohols, aromatic compounds, fatty acids, fatty acid esters, ethers, phthalates, phenols, pyridines, steroids, alkaloids, monoterpenes, sesquiterpenes, diterpenes, tetraterpenes, and their derived compounds were also identified and reported from the EOs profiles of these plants from different areas/countries [5–7,10,11,72–78].

There are variations and slight similarity in the contents and chemical constituents or classes of compounds of the EOs obtained from the same plant organs or among the species of the genus *Taxus*. The most frequent components with high concentrations of this genus are 1-octen-3-ol, *cis*-3-hexen-1-ol (aliphatic unsaturated alcohols), caryophyllene oxide (oxygenated sesquiterpene), myrtenol (oxygenated monoterpene), elemicin (phenylpropanoid), *trans*-2-hexenal (aldehyde), α-pinene (monoterpene hydrocarbon), and laminitol (cyclic polyhydroxy alcohol) (Table 2). The structures of these chemical compounds are appeared in Figure 4. Of these compounds, 1-octen-3-ol was detected to be a predominant compound of the EO isolated from *T. canadensis* of Canada [2], *T. baccata* growing in Turkey [4], Serbia [5], and Netherlands [1]. *cis*-3-Hexen-1-ol was also predominant in the EO obtained by simultaneous distillation extraction using diethyl ether as a solvent from *T. wallichiana* var *mairei* from China [3]. Elemicin was the most abundant volatile component in the oil obtained from the sapwood and bark of *T. chinensis* and from heartwood extracted using ethanol and methanol as solvent [6]. The highest content of α-pinene was also found in the EOs isolated from *T. chinensis* stems [7], leaves, and woods [8]. However, the oil isolated from the heart wood of the same plant by using ethanol and benzene as solvent was characterized by high amount of laminitol [6].

On the other hand, the fatty acid (FA) compositions of oils extracted from *Taxus* species with different methods showed that they constitute many saturated and unsaturated (both monounsaturated and polyunsaturated) fatty acid compounds. Palmitic acid and oleic acid were identified as the most predominant components of saturated fatty acid (SFA) and monounsaturated fatty acid (MUFA), respectively, whereas linoleic acid was reported as a principal compound of polyunsaturated fatty acid (PUFA) followed by taxoleic and α-linolenic acids. All these saturated and unsaturated FAs were frequently reported as major fatty acid compositions from the oils of plants of the genus *Taxus* from different regions. However, the concentrations of the FAs and the overall fatty acids profiles of these oils showed variations. Oleic acid was identified as the most abundant component (20.87%) of the oil extracted from *T. baccata* leaves of Iran [11]. The oils isolated from the fresh and dried needles of the same plant of Turkey were dominated mostly (19.6%–22.5%) by palmitic acid [10].

The major fatty acid in the oil obtained from the red arils of this plant collected from Zielona Gora, Poland was linoleic acid (30.92%), followed by palmitic (20.43%), α-linolenic (18.53%), myristic (9.84%), and oleic (9.52%) acids. However, α-linolenic acid was the most abundant (23.43%–26.50%) fatty acid component of the same part of this plant collected from Warsaw, Koszalin and Cracow sites, Poland, followed by palmitic (22.37%–24.37%) and linoleic (19.40%–21.33%) acids [9]. The seeds oil of *T. chinensis*, *T. canadensis*, *T. cuspidata*, and *T. baccata* mainly composed of oleic (34.31%–59.3%), linoleic (16.8%–34.22%) and taxoleic (9.5%–16.16%) acids [12–15]. Other MUFAs such as *cis*-vaccenic (36.73 – 36.96%) and *trans*-palmitoleic (23.66%–24.05%) acids together with palmitic acid (6.19%) were also reported as the most predominant compositions of the oils isolated from *T. chinensis* var. *mairei* leaves [77]. All the variations in the contents and compositions of the oils of *Taxus* species may be due to different factors including extraction and analysis methods [66,69–71].

### 1.2. Biological activities of oils of Taxus species

There is a shortage of literature on the biological activities of oils of *Taxus* species. However, the previously reported results on the investigated antimicrobial activities of the oils of these plants evaluated using MIC, MBC, ZI, and IC_50_ approaches against pathogenic yeast, *Candida albicans* and various gram (+) and gram (−) bacteria are presented in Table 3. Table 4 also represents the compilation of the results on their antioxidant activities. Among all *Taxus* plants from different regions, only the oils of *T. chinensis*, *T. cuspidata*, *T. chinensis* var. *mairei*, and *T. media* were evaluated for their biological activities.

Almost all the investigated oils displayed strong antimicrobial activity toward the tested strains of bacteria and a fungus. In general, these oils are more susceptible toward gram (+) bacteria than gram (−) ones. The oil from *T. chinensis* leaves with high amount of α-pinene showed the highest antibacterial activity with MIC value of 16.0 μg/mL as well as IC_50_ value of 3.98 μg/mL against *E. faecalis* (gram-positive bacterium). This same oil also demonstrated potent activity towards a fungus, *C. albicans* (MIC = 128.0 *μg*/mL, IC_50_ = 55.67 *μg*/mL) and other gram-positive bacteria such as *B. cereus* (MIC = 64.0 *μg*/mL, IC_50_ = 19.78 *μg*/mL) and *S. aureus* (MIC = 256.0 *μg*/mL, IC_50_ = 100.56 *μg*/mL), but no activity towards the gram (−) pathogens like *S. enterica*, *E. coli*, and *P. aeruginosa*. The oil obtained from the woods of the same plant with high content of α-pinene also displayed strong antimicrobial activities against *E. faecalis* (MIC = 64.0 *μg*/mL, IC_50_ = 20.33 *μg*/mL), *S. aureus* (MIC = 128.0 *μg*/mL, IC_50_ = 56.78 *μg*/mL), *E. coli* (MIC = 256.0 *μg*/mL, IC_50_ = 87.78 *μg*/mL), and *C. albicans* (MIC = 256.0 *μg*/mL, IC_50_ = 89.67 *μg*/mL). However, this oil showed no activity towards *P. aeruginosa* and *S. enterica* [8]. The powerful antimicrobial activities of these oils are probably related to the high content of α-pinene. This compound was reported to have antimicrobial activities [66,79].

Bajpai et al. [74] also reported the good bactericidal potential of the leaves oil of *T. cuspidata*. This oil was very active against *B. cereus* (ZI = 34.0 mm, MIC = 250 *μg*/mL, MBC = 500 *μg*/mL), *S. aureus* (ZI = 34.0 mm, MIC = 250 *μg*/mL, MBC = 500 *μg*/mL), *L. monocytogenes* (ZI = 27.0 mm, MIC = 500 *μg*/mL, MBC = 1000 *μg*/mL), *E. coli* (ZI = 24.0 mm, MIC = 500 *μg*/mL, MBC = 1000 *μg/*mL) and *S. typhimurium* (ZI = 22.0 mm, MIC = 500 *μg*/mL, MBC = 1000 *μg*/mL). The antibacterial activity of the leaves oil of *T. media* was stronger than that of *T. chinensis* var. *mairei* leaves oil [73]. The MIC values for the oil of *T. chinensis* var. *mairei* for *S. aureus* and *E. coli* were 98% and 95%, respectively. However, the values for the oil of *T. media* on these bacteria were both 5%. These oils demonstrated high activity to *E. coli* in comparison to *S. aureus*. The different chemical compositions and their percentages of the oils are most likely responsible for the different properties found towards the microbes.

According to the literature survey, the antioxidant activities of only the oil of *T. cuspidata* fresh stems have been investigated and reported. To determine the activities of the oil, antioxidant assays such as DPPH, reducing power activity, lipid peroxidation, nitric oxide, superoxide, and hydroxyl radicals were employed. In Table 4, results of these activities of the oil of this *Taxus* plant are shown. The results demonstrated that the oil exhibited powerful antioxidant activity in DPPH assay with an inhibitory effect of 92.8% at 500 *μg*/mL concentration. At 100 *μg*/mL, the inhibitory effects of α-tocopherol and ascorbic acid standards were 73.4% and 72.9%, respectively. The oil also had strong inhibitory effects (71.7%, 73.7%, and 80.0%) which were comparable to the standards on superoxide, hydroxyl, and nitric oxide radicals, respectively (Table 4). The inhibitory effects of α-tocopherol and ascorbic acid were 74.4% and 73.0% on superoxide radicals, whereas BHA and ascorbic acid were 70% and 73.3% on hydroxyl radicals, respectively. Moreover, the oil showed better lipid peroxidation inhibition (80.2%) than the standards, α-tocopherol (80.1%) and BHA (76.5%) all at 250 *μg*/mL concentration. The same oil also exhibited significant reducing power activity (absorbance value, 1.1) in comparison to the reference compounds, α-tocopherol (absorbance value, 1.1) and ascorbic acid (absorbance value, 1.2) at 25 *μg*/mL concentration. The strong antioxidant property of the oil was due to the existence of phenolic compounds such as umbelliferon and eugenol and fatty acids in the oil [76]. The volatile chemical constituents of the leaves of *T. chinensis* var. *mairei* were also proved to be used as natural and supplementary reagents to treat hypertension [38].

## 2. Factors in the regeneration of the endangered Taxus plants

As discussed in detail in Section 1, *Taxus* species have a variety of medicinal and economic values; their oils also have several biological activities and bioactive chemical constituents. However, they are highly endangered plants principally due to their high demand for the extraction of taxol drug [81] and regeneration of these plants have been of large concern worldwide [20]. The seeds of *Taxus* species are highly dormant and due to this, they are extremely difficult to germinate. These and other factors such as low seed production, slow growth, overexploitation, lack of awareness, narrow range, slow propagation, destructive harvesting, habitat specificity, high value, climate change, habitat loss or destruction, over-grazing and changes in forest management were the reasons identified by several researchers why *Taxus* plants face extinction and need urgent conservation [17,20,36,37,81]. All these diverse factors have negative impact on the anatomy, physiology, and behavioral peculiarities of yews that ultimately impact their regeneration. In this review paper, in this section, the major biotic and abiotic factors that limit the regeneration and growth of these important and useful but endangered plants are explained in detail based on the data collected from several research papers and the literature.

### 2.1. Climate change and temperature effects

Various climatic and environmental factors can affect the distribution and regeneration of *Taxus* species in the forest [36]. In addition to fungi, insects, viruses, bacteria, rodents, and pests; climate change and disturbances from fires have a significant impact on the establishment, growth, and spread of these plants [36,81,82]. High temperatures and their variations have a direct influence on the conditions for the growth and development of these plants [83]. Forest fires are one of the major causes for the increased temperatures in the forests worldwide [84]. The high temperatures negatively affect the plant regeneration, mostly in the southern aspect resulting in excessive loss of moisture due to an increase in evapotranspiration [83]. Losses of several plant species worldwide have been attributed to temperature fluctuations [85]. Climate change is one of the major problems of the 21st century [86]. Climatic changes due to temperature variation have also been reported to result in decreased pollination and seed production [87]. Thus, climate change and the drought occurrences because of these changes also have negative effects on the regeneration of *Taxus* plants [36,81] because the rate of growth and survival of the seedlings of yews could be determined on the basis of their resistance to these and the aforesaid destructive components of the environment as well as climate shocks and events [36,88].

### 2.2. Canopy closure

Local environmental conditions can also affect the germination of the seeds of the endangered *Taxus* species [89]. The seeds of these plants germinate in the shady areas under the canopy of the trees than in canopy gaps [90,91]. Most seedlings which are found under the mother trees of their geographical sites clearly indicate the requirement of minimal light for the germination of the seeds of *Taxus* species and their regeneration potential on deep shady, moist, and sheltered sites [90]. However, the availability of light is necessary for regeneration [88]. The stand structure and canopy cover have played a major role in the establishment of the seedlings of *Taxus* plants [91]. Due to these effects, the rates of the establishment of the seedlings were very low and hence influence the regeneration and vitality of the plants [92]. Although *Taxus* plants are known to thrive under dense forest canopy for a long time in the seedling stage, at maturity, they need canopy gaps without which they may lose the competition for essential resources [91]. It has been reported that a higher percentage of the living crown of associated species can harm *Taxus* species formation [93]. Sometimes, herbivores also play a key role in the development of canopy gaps [82,93,94]. Hence, good regeneration or survival of *Taxu*s plants is dependent on the suitability of the local environments.

### 2.3. Herbivores

Herbivores (insects, deer, rabbit, moose, rodents, goats, horses, cattle, sheep, and others) adversely affect the regeneration of plants particularly concerning the overall growth of seedlings and saplings, their proliferation and attainment of luxuriance [82,93,95]. As compared to healthy plants, in the plants damaged by the herbivores, besides the overall plant height pollination, seed production and stand structural dynamism are significantly different. One of the reasons for the poor regeneration of plants of the genus *Taxus* has been attributed to the damage caused by the abovementioned grazing animals [93,95,96]. The immense browsing pressure of the plants by these grazing animals sometimes even proves lethal to their establishment, growth, and development because the animals readily eat the seedlings as well as the needles/leaves, buds, shoots, and bark of *Taxus* trees [82,94–99]. In some areas, the seeds of these plants along with their red arils were also eaten by monkeys, rats, birds (especially *Turdu*s species), and children [36,90,97]. Thus, herbivory can also be the main factor influencing the growth, development, and regeneration of yews.

### 2.4. Availability of water and species competition

The availability of water in the forest in the areas where *Taxus* plants are found can also play a great role in their regeneration. As reported by the researchers, there is a scarcity of water in the temperate regions of the southern aspect harboring natural habitats such as forests, which is a major constraint in the regeneration of these plants while northern aspects are impacted more by shade [83,88]. In general, landscapes with more availability of water, humidity and rainfall have a higher density of regeneration in comparison to drier places at both regional as well as continental scales [88,100]. Thus, regeneration of *Taxus* plants is closely associated to an abiotic factor, water availability. Moreover, *Taxus* plants strongly face competition for light, nutrients, and water availability with other plants or the same species that decrease the numbers of their populations by affecting the seedlings’ survival rates [82,91]. Hence, the availability of sufficient water resources and protecting the plants from other competing species are obligatory requirements for saving *Taxus* species from getting into a more endangered status and also preventing fragmentation into small as well as marginal populations.

### 2.5. Dispersal of seeds

The dispersals of seeds of plants can also play an important role in their regeneration. The dispersal of seeds in *Taxus* species is a pivotal phenomenon due to unsuitable microsites and the role of predators in seed dispersion phenomena [101]. The seeds of these plants are dispersed to unfavorable sites mainly by birds and monkeys [36,82,89,90,98]. They are also not able to survive if dispersed in the places that are cleared for the purposes of agricultural activities [36]. This is because the dispersed seeds of *Taxus* plants have been reported to be highly dormant and hard to germinate [98]. During the postdispersal stage, the seeds can also be destroyed by rodents [90,98,99]. The rodent populations in the forest are quite high and they eat seeds of *Taxus* species, which significantly reduces the chances for regeneration and contributes to low numbers of seedlings [97]. Not only rodents, birds, and monkeys, but also humans are equally responsible in this regard [92,99]. Moreover, in their natural conditions and inside their geographical ranges, the ripe seeds of *Taxus* plants dispersed in autumn and in the late summer do not germinate before the second spring, and germinate in the next spring or maybe later [89,93]. Furthermore, the geostatistical investigation has demonstrated that seedlings that grow in patches in the forest areas avoid their direct competition with mature trees for resource mobilization [102]. Thus, the dispersal of seeds is also a factor that strongly affects the regeneration of the endangered yews.

### 2.6. Anthropogenic disturbances

Nowadays, anthropogenic activities are playing a significant role in the decline of *Taxus* species populations [103]. These activities are closely related to agricultural practices, destruction of habitats, deforestation, fuel, lopping, regular removal of bark, overexploitation, and unsustainable extraction and burning [36,88,90,92,103]. They are major reasons that highly affect the growth and regeneration of these endangered plants. Of all these human disturbances, overexploitation of the bark and leaves of *Taxus* species for pharmaceutical uses are listed as primary reasons for their unsustainable regeneration [19]. Overharvesting of plant parts for domestic purposes has also brought the plants under severe threat [22]. Additionally, browsing and bark peeling by domestic cattle adversely affect the growth of seedlings, saplings, and their vitality [99]. Therefore, not only climatic changes and all the abovementioned factors, but also anthropogenic disturbances play a detrimental role in the proliferation of the population of yews in their region [83,104].

In summary, all the abovementioned major biotic and abiotic factors are bringing plants of the genus *Taxus* to severe endangerment. Therefore, to protect these natural wild resources, urgent conservation actions must be taken for all of the plants in their region. Some of these conservation actions include building fences for the protection of *Taxus* plant’s natural regeneration, protecting them by guards, raising awareness in local people, and limiting the big game hunting of the ungulates to reduce their population. In addition, the forest managements can also save the older or matured *Taxus* trees because they are sources of seeds that can ensure the regeneration of other *Taxus* trees and also maintain the ecological integrity of these plants stands. *Taxus* plants are very sensitive at their seedling stage and protecting the seedlings from grazing damage and browsing is also needed for the growth, establishment, and regeneration of these plants. Artificial regenerations of *Taxus* plants from their seeds are extremely poor because of the hard-coated seeds, and the growth and development of the seedlings are very slow [20]. Therefore, tissue, hairy root, cell, and other organ cultures technology by specialists, reported as very fast, effective, and successful tools for the regeneration and propagation of plants [18,81] are required as an alternative technique to save *Taxus* plants from extinction and endangerment. This technique is very helpful for the production of a high concentration of taxol and its precursors and other important secondary metabolites from *Taxus* trees without destroying them [18,105].

## 3. Conclusions

*Taxus* is the largest genus of the family Taxaceae and comprises about 24 species with 55 varieties, distributed mainly in Asia, Europe, North Africa, and North America. Its species gained global recognition for their anticancer drug taxol. *Taxus* species are also used to relieve edema, to remove toxicity from the body, and to treat diseases like lung disorders, epilepsy, nervousness, hysteria, malaria, nephropathy, and diabetic nephropathy. They are reported to exhibit antileukemic, analgesic, cytotoxic, antiinflammatory, sedative, anticancer, anticonvulsant, antipyretic, antibacterial, antimitotic, tranquilizing, antifungal, and antiseptic properties. According to the literature, among the identified plants of the genus *Taxus*, only eight of them, namely *T. baccata*, *T. chinensis*, *T. canadensis*, *T. media*, *T. cuspidata*, *T. wallichiana*, *T. wallichiana* var *mairei*, and *T. chinensis* var. *mairei*, were studied concerning the chemical constituent and only four, such as *T. chinensis*, *T. cuspidata*, *T. chinensis* var. *mairei*, and *T. media*, have been studied in terms of biological activities (only antifungal, antibacterial, antioxidant, and antihypertensive activities) of their oils. Generally, essential oils of the investigated *Taxus* species were dominated mostly by alcohols. The most frequent components with high concentrations of these essential oils are *cis*-3-hexen-1-ol, 1-octen-3-ol, caryophyllene oxide, myrtenol, elemicin, *trans*-2-hexenal, α-pinene, and laminitol. Palmitic, oleic, linoleic, taxoleic, and α-linolenic acids were the most predominant and frequently reported fatty acid constituents of the oils (lipids) of *Taxus* plants from different regions. The oils of the investigated plants of the genus *Taxus* have demonstrated powerful antifungal, antibacterial, antioxidant and antihypertensive activities. However, the species of this genus are the most threatened and endangered plants in their geographical ranges. Various biotic and abiotic factors are affecting the survival of these precious species and due to these, their regeneration is very poor. Of these, climatic and environmental factors and anthropogenic disturbances are the main reasons for the poor regeneration. Therefore, to protect plants of the genus *Taxus*, urgent conservation actions must be taken by forest managers, local communities, governments and other stakeholders for all of the plants in their region. In the future, studies are also needed in the researches of pharmacists, chemists, biologists, and phytochemists to investigate the chemical constituents and biological activities of oils of the unstudied and less studied *Taxus* plants and foresters, ecologists, and environmentalists regarding their most effective regeneration.

## Figures and Tables

**Table 1:** Content of taxol and other taxoids (precursors to taxol) in different parts of *Taxus* species.

*Taxus* species	Country	Plant part	Content	References
*T. baccata*	Poland	Needles	Taxol: 0.011% dw	[54]
	France		Taxol: 0.0005%–0.0184% dw	[55]
			10 DAB III: 0%–0.099% dw	
	Britain		Taxol: 0.0055%–0.0065% dw	
			10 DAB III: 0.062%–0.073% dw	
	Switzerland		Taxol: 0.0072% dw	
			10 DAB III: 0.054% dw	
	Germany		Taxol: 0.0008%–0.0186% dw	
			10 DAB III: 0.0003%–0.075% dw	
	Ireland		Taxol: 0.00064%–0.0115% dw	
			10 DAB III: 0.00292%–0.08828% dw	
	Georgia	Twigs and leaves	Taxol: 0.0057%–0.0122% dw	[50]
			Cephalomannine: 0.0032%–0.0067% dw	
			Baccatin III: 0.0044%–0.0088% dw	
	Russia		Taxol: 0.0033%–0.0125% dw	
			Cephalomannine: 0.0018%–0.0079% dw	
			Baccatin III: 0.0022%–0.0097% dw	
	Ukraine		Taxol: 0.0018%–0.0151% dw	
			Cephalomannine: 0.0007%–0.0104% dw	
			Baccatin III: 0.0022%–0.0134% dw	
		Bark	Taxol: 0.0148% dw	
	USA	Stems	Taxol: 0.001% dw	[51]
		Twigs	Taxol: 0.0006% dw	
		Leaves	Taxol: 0.003% dw	
*T. baccata* (female)	Hungary	Foliage	Taxol: 0.0029%	[48]
*T. baccata* (male)	Hungary	Bark	Taxol: 0.0061%	
*T. baccata* “a” sign male clone	Hungary	Foliage	Taxol: 0.0027%	
*T. baccata* “b” sign clone	Hungary	Bark	Taxol: 0.0040%	
*T. baccata* “c” sign clone	Hungary	Bark	Taxol: 0.0029%	
*T. baccata* “d” sign clone	Hungary	Bark	Taxol: 0.0068%	
*T. baccata* “e” sign clone	Hungary	Bark	Taxol: 0.0093%	
*T. baccata* L.	Poland	Needles	Taxol: 0.0105% dw	[54]
			Taxol: 0.00251% dw**	[56]
			Cephalomannine: 0.00139% dw**	
			Taxol: 0.00194% dw***	
			Cephalomannine: 0.00102% dw***	
		Twigs	Taxol: 0.0016% dw **	
			Cephalomannine: 0.0004% dw**	
			Taxol: 0.00187% dw ***	
			Cephalomannine: 0.00055% dw***	
		Surface of twigs	Taxol: 0.00000084% fw	[57]
			Baccatin III: 0.00000258% fw	
			10 DAB III: 0.00000148% fw	
			Cephalomannine: n.d.	
		Needles	Taxol: 0.00117%–0.00337% dw	
			Baccatin III: 0.00193%–0.00453% dw	
			10 DAB III: 0.00502%–0.01459% dw	
			Cephalomannine: 0.00346%–0.02048% dw	
		Stems	Taxol: 0.00023%–0.00189% dw	
			Baccatin III: 0.00091%–0.00471% dw	
			10 DAB III: 0.0068%–0.03022% dw	
			Cephalomannine: 0.00062%–0.00528% dw	
		Leaves	Taxol: 0.01167%	[52]
		On the surface of the needles	Taxol: 0.00001%	
	Netherlands and UK	Needles	Taxol: 0.0041% dw	[53]
			Cephalomannine: 0.0022% dw	
			Baccatin III: 0.0014% dw	
			10 DAB III: 0.0762% dw	
	Poland	Red arils	Taxol: 0.000002% dw^Z^	[9]
			Cephalomannine: 0.000005% dw^Z^	
			Baccatin III: 0.00063% dw^Z^	
			10 DAB III: 0.00198% dw Z	
			Taxol: 0.00001% dw^W^	
			Cephalomannine: 0.000018% dw^W^	
			Baccatin III: 0.0002% dw^W^	
			10 DAB III: 0.00039% dw^W^	
			Taxol: 0.000005% dw^K^	
			Cephalomannine: 0.000012% dw^K^	
			Baccatin III: 0.00023% dw^K^	
			10 DAB III: 0.00074% dw^K^	
			Taxol: 0.000005% dw^C^	
			Cephalomannine: 0.000012% dw^C^	
			Baccatin III: 0.00024% dw^C^	
			10 DAB III: 0.00041% dw^C^	
*T. baccata* basic species	Hungary	Foliage	Taxol: 0.0146%	[48]
*T. baccata “Adpressa”*	Hungary	Bark	Taxol: 0.0047%	
	Ireland	Needles	Taxol: 0.00762% dw	[55]
			10 DAB III: 0.01674% dw	
	France		Taxol: 0.0012%–0.0023% dw	
			10 DAB III: 0.01368%–0.0663% dw	
*T. baccata adpressa aurea*	France	Needles	Taxol: 0.0005%–0.0005% dw****	
			10 DAB III: 0%–0.002759% dw	
*T. baccata “’Aurea”*	Hungary	Foliage	Taxol: 0.0025%–0.0053%	[48]
		Bark	Taxol: 0.0056%	
	Ireland	Needles	Taxol: 0.0101% dw	[55]
			10 DAB III: 0.02382% dw	
	France		Taxol: 0.0018%–0.004% dw	
			10 DAB III: 0.00458%–0.02004% dw	
*T. baccata Barronii*	France	Needles	Taxol: 0.0035%–0.0051% dw	
			10 DAB III: 0.02101%–0.02162% dw	
*T. baccata “Dovastoniana”*	Hungary	Foliage	Taxol: 0.0071%	[48]
	Ireland	Needles	Taxol: 0.00736% dw	[55]
			10 DAB III: 0.01011% dw	
*T. baccata “Elegantissima”*	Hungary	Foliage	Taxol: 0.0029%	[48]
	Poland	Needles	Taxol: 0.017% dw	[54]
			Taxol: 0.00299% dw**	[56]
			Cephalomannine: 0.00271% dw**	
			Taxol: 0.00244% dw***	
			Cephalomannine: 0.002% dw***	
		Twigs	Taxol: 0.00086% dw **	
			Cephalomannine: 0.00039% dw**	
			Taxol: 0.00063% dw ***	
			Cephalomannine: 0.00035% dw***	
	Ireland	Needles	Taxol: 0.00316% dw	[55]
			10 DAB III: 0.009% dw	
	Poland	Leaves	Taxol: 0.002591%	[52]
		On the surface of needles	Taxol: 0.000015%	
*T. baccata erecta*	Ireland	Needles	Taxol: 0.00848% dw	[55]
			10 DAB III: 0.009% dw	
*T. baccata “Fastigiata”*	Hungary	Foliage	Taxol: 0.0027%–0.01%	[48]
	France	Needles	Taxol: 0.0041%–0.0142% dw	[55]
			10 DAB III: 0.00462%–0.04179% dw	
	Ireland		Taxol: 0.00475%–0.019% dw	
			10 DAB III: 0.01253%–0.04542% dw	
*T. baccata “Fastigiata” “Aurea “*	Hungary	Bark	Taxol: 0.0023%–0.0037%	[48]
	France	Needles	Taxol: 0.0021%–0.0099% dw	[55]
			10 DAB III: 0.01298%–0.04439% dw	
*T. baccata fastigiata aurea marginata*	France	Needles	Taxol: 0.0015%–0.0028% dw	
			10 DAB III: 0.01005%–0.01179% dw	
*T. baccata fructolutea*	Ireland	Needles	Taxol: 0.00929% dw	
			10 DAB III: 0.0233% dw	
*T. baccata glauca*	Ireland	Needles	Taxol: 0.00489% dw	
			10 DAB III: 0.017% dw	
*T. baccata “Lutea”* (female)	Hungary	Bark	Taxol: 0.0179%	[48]
*T. baccata marginata aurea*	France	Needles	Taxol: 0.0024%–0.0043% dw	[55]
			10 DAB III: 0.01722%–0.02674% dw	
*T. baccata “Overeyndenri”*	Hungary	Bark	Taxol: 0.0024%–0.0079%	[48]
*T. baccata “Repanda”*	Hungary	Bark	Taxol: 0.0048%	
*T. baccata ‘Repandens’*	France	Needles	Taxol: 0.0012%–0.0034% dw	[55]
			10 DAB III: 0.02302%–0.04125% dw	
	USA		Taxol: 0.003% dw	[58]
			10 DAB III: 0.02% dw	
		Stems	Taxol: 0.001% dw	
			10 DAB III: n.q.	
*T. baccata “Semperaurea”*	Hungary	Bark	Taxol: 0.0049%	[48]
	France	Needles	Taxol: 0.0054%–0.0067% dw	[55]
			10 DAB III: 0.01869%–0.0272% dw	
*T. baccata variegata*	France	Needles	Taxol: 0.0007%–0.0038% dw	
			10 DAB III: 0.00823%–0.013% dw	
*T. brevifolia*	Netherlands and UK	Needles	Taxol: 0.013% dw	[53]
			Cephalomannine: 0	
			Baccatin III: 0.0296% dw	
			10 DAB III: 0.0041% dw	
	Hungary	Bark	Taxol: 0.0048%	[48]
	USA		Taxol: 0.02%–0.06% dw	[59]
			10 DAB III: 0.03%–0.03% dw*****	
	Ireland	Needles	Taxol: 0.00116% dw	[55]
			10 DAB III: 0.013%–0.014% dw	
	France		Taxol: 0.0008%–0.0015% dw	
			10 DAB III: 0.00774%–0.02976% dw	
	USA and Canada	Bark	Taxol: 0.015% dw	[51]
		Roots	Taxol: 0.004% dw	
		Wood	Taxol: 0.0006% dw	
		Wood with Bark	Taxol: 0.0003% dw	
		Branches	Taxol: 0.0017% dw	
		Leaves/needles	Taxol: 0.0015% dw	
		Twigs	Taxol: 0.0012% dw	
		Seedlings	Taxol: 0.0058% dw	
	USA	Shoots	Taxol: 0.001%–0.033% dw	[60]
		Bark	Taxol: 0.001%–0.013% dw	
			Cephalomannine: 0.002%–0.027% dw	
			Baccatin III: 0.001%–0.050% dw	
		Needles	Taxol: 0.001%–0.003% dw	
			Cephalomannine: 0.002%–0.008% dw	
			Baccatin III: 0.013%–0.030% dw	
			Taxol: 0.006% dw	[58]
			10 DAB III: 0.01% dw	
*T. canadensis*	Netherlands and UK	Needles	Taxol: 0.0285% dw	[53]
			Cephalomannine: 0.0289% dw	
			Baccatin III: 0.0224% dw	
			10 DAB III: 0.2665% dw	
	Ireland		Taxol: 0.00158% dw	[55]
			10 DAB III: 0.016% dw	
	France		Taxol: 0.0036%–0.0046% dw	
			10 DAB III: 0.02919%–0.04753% dw	
	Canada		Taxol: 0.00975%–0.01561% dw	
			10 DAB III: 0.02818%–0.04279% dw	
	USA		Taxol: 0.009% dw	[58]
			10 DAB III: 0.002% dw	
		Stems	Taxol: 0.002% dw	
			10 DAB III: 0.005% dw	
	Hungary	Foliage	Taxol: 0.0095%	[48]
*T. celebica*	Netherlands and UK	Needles	Taxol: 0.0026% dw	[53]
			Cephalomannine: 0	
			Baccatin III: 0	
			10 DAB III: 0.007% dw	
*T. chinensis*	China	Needles	Taxol: 0.0039%	[61]
			Cephalomannine: 0.0112%	
			10 DAB III: 0.0168%	
		Leaves	Taxol: 0.0088% dw	[49]
			Cephalomannine: 0.0058% dw	
	Ireland	Needles	Taxol: 0.00286% dw	[55]
			10 DAB III: 0.006% dw	
	China		Taxol: 0.01135%	[62]
			Cephalomannine: 0.00899%	
			10 DAB III: 0.00559%	
			Baccatin III: 0.00338%	
*T. cuspidata*	China	Needles	Taxol: 0.005%	[61]
			10 DAB III: 0.0046%	
			Cephalomannine: 0.0093%	
	Netherlands and UK		Taxol: 0.0105% dw	[53]
			Cephalomannine: 0.004% dw	
			Baccatin III: 0.0015% dw	
			10 DAB III: 0.012% dw	
		Stem bark	Taxol: 0.013%–0.017% dw	[49]
			Cephalomannine: 0.0080%–0.032% dw	
	Hungary	Foliage	Taxol: 0.0037%	[48]
	Ireland	Needles	Taxol: 0.00728% dw	[55]
			10 DAB III: 0.002% dw	
	France		Taxol: 0.0008%–0.0169% dw	
			10 DAB III: 0%–0.05319% dw	
	Roumania		Taxol: 0%–0.00186% dw	
			10 DAB III: 0%–0.02493% dw	
	USA	Twigs	Taxol: 0.0006% dw	[51]
	China	Needles	Taxol: 0.00996%	[62]
			Cephalomannine: 0.02486%	
			10 DAB III: 0.00277%	
			Baccatin III: 0.00254%	
*T. cuspidata ‘Capitata’*	USA	Needles	Taxol: 0.008% dw	[58]
			10 DAB III: 0.002% dw	
		Stems	Taxol: 0.004% dw	
			10 DAB III: 0.002% dw	
*T. cuspidata* Sieb. et Zucc.	China	Stem bark*	Taxol: 0.031% dw	[49]
			Cephalomannine: 0.023% dw	
		Root bark*	Taxol: 0.018% dw	
			Cephalomannine: 0.018% dw	
		Fibrous roots*	Taxol: 0.014% dw	
			Cephalomannine: 0.010% dw	
		Twigs and leaves*	Taxol: 0.0059% dw	
			Cephalomannine: 0.0055% dw	
	Poland	Needles	Taxol: 0.0105% dw	[54]
			Taxol: 0.0181% dw**	[56]
			Cephalomannine: 0.00309% dw**	
			Taxol: 0.01284% dw***	
			Cephalomannine: 0.00286% dw***	
		Twigs	Taxol: 0.00036% dw **	
			Cephalomannine: 0.00019% dw**	
			Taxol: 0.00027% dw ***	
			Cephalomannine: 0.00024% dw***	
		Leaves	Taxol: 0.04643%	[52]
		On the surface of needles	Taxol: 0.000118%	
*T. floridana*	Ireland	Needles	Taxol: 0.0076% dw	[55]
			10 DAB III: 0.003% dw	
	Netherlands and UK		Taxol: 0.0516% dw	[53]
			Cephalomannine: 0	
			Baccatin III: 0	
			10 DAB III: 0.1689% dw	
*T. globosa*	Netherlands and UK	Stems	Taxol: 0.0064%	[63]
		Cortex	Taxol: 0.0085%	
		Needles	Taxol: 0.0130%	
			Taxol: 0.0433% dw	[53]
			Cephalomannine: 0.048% dw	
			Baccatin III: 0.0168% dw	
			10 DAB III: 0.1395% dw	
*T. hunevelliata*	Hungary	Foliage	Taxol: 0.0032%	[48]
*T. x hunnewelliana*	France	Needles	Taxol: 0.0083%–0.0104% dw	[55]
			10 DAB III: 0%–0.00867% dw	
	Netherlands and UK		Taxol: 0.0041% dw	[53]
			Cephalomannine: 0	
			Baccatin III: 0	
			10 DAB III: 0.0063% dw	
*T. mairei*	China	Leaves	Taxol: 0.0069%–0.0127% dw	[64]
*T. x media*	Hungary	Foliage	Taxol: 0.0036%	[48]
	Poland	Needles	Taxol: 0.036% dw	[54]
	USA	Stems	Taxol: 0.002% dw	[51]
		Twigs	Taxol: 0.009% dw	
		Leaves	Taxol: 0.002% dw	
	China	Needles	Taxol: 0.01301%	[62]
			Cephalomannine: 0.00715%	
			10 DAB III: 0.00875%	
			Baccatin III: 0.00405%	
			Taxol: 0.0051%	[61]
			10 DAB III: 0.0132%	
			Cephalomannine: 0.0122%	
*T. x media Brownii*	France	Needles	Taxol: 0.0041%–0.0064% dw	[55]
			10 DAB III: 0.007%–0.03316% dw	
*T. x media ‘Densiformis’*	France	Needles	Taxol: 0.004%–0.007% dw	
			10 DAB III: 0.0078%–0.03202% dw	
	USA		Taxol: 0.002% dw	[58]
			10 DAB III: 0.007% dw	
		Stems	Taxol: 0.003% dw	
			10 DAB III: 0.002% dw	
*T. x media* var. *Hatfieldii*	Poland	Needles	Taxol: 0.02% dw	[54]
			Taxol: 0.00128% dw**	[56]
			Cephalomannine: 0.00043% dw**	
			Taxol: 0.0013% dw***	
			Cephalomannine: 0.00048% dw***	
		Twigs	Taxol: 0.00201% dw **	
			Cephalomannine: 0.00045% dw**	
			Taxol: 0.00211% dw ***	
			Cephalomannine: 0.00056% dw***	
	France	Needles	Taxol: 0.0087%–0.0115% dw	[55]
			10 DAB III: 0.00393%–0.01008% dw	
	Poland	Leaves	Taxol: 0.04852%	[52]
		On the surface of needles	Taxol: 0.00008%	
*T. x media “Hicksii”*	Hungary	Foliage	Taxol: 0.0056%	[48]
		Bark	Taxol: 0.0031%	
	Poland	Needles	Taxol: 0.015%–0.02% dw	[54]
			Taxol: 0.00658% dw**	[56]
			Cephalomannine: 0.0047% dw**	
			Taxol: 0.0054% dw***	
			Cephalomannine: 0.00403% dw***	
		Twigs	Taxol: 0.00236% dw **	
			Cephalomannine: 0.0022% dw**	
			Taxol: 0.00183% dw ***	
			Cephalomannine: 0.00162% dw***	
	Britain	Needles	Taxol: 0.00507%–0.0069% dw	[55]
			10 DAB III: 0.0487%–0.08754% dw	
	France		Taxol: 0.0109%–0.0112% dw	
			10 DAB III: 0.00418%–0.03025% dw	
	USA		Taxol: 0.01% dw	[58]
			10 DAB III: 0.009% dw	
		Stems	Taxol: 0.005% dw	
			10 DAB III: 0.002% dw	
	Poland	Leaves	Taxol: 0.08859%	[52]
		On the surface of the needles	Taxol: 0.000129%	
*T. x media stricta viridis*	France	Needles	Taxol: 0.0049%–0.0088% dw	[55]
			10 DAB III: 0.01045%–0.0134% dw	
*T. wallichiana*	Pakistan	Leaves	Taxol: 0.018%–0.022 wt %	[40]
		Stem	Taxol: 0.005%–0.006 wt %	
		Bark	Taxol: 0.049%–0.066 wt %	
		Root	Taxol: 0.023%–0.087 wt %	
	India	Stem bark	Taxol: 0.011%–0.043% dw	[65]
			Baccatin III: 0.38%–3.44% dw	
			10 DAB III: 0.081%–0.704% dw	
		Needle leaves	Taxol: 0.016%–0.031% dw	
			Baccatin III: 0.065%–1.442% dw	
			10 DAB III: 0.015%–0.621% dw	
		Stems	Taxol: 0.001%–0.012% dw	
			Baccatin III: 0.011%–0.382% dw	
			10 DAB III: 0.035%–0.454% dw	
		Bark****	Taxol: 0.064%–8.032 g/plant dw	[42]
		Bark of male trees	Taxol: 0.0376–0.1167%	
		Bark of female trees	Taxol: 0.0129–0.0810%	
	India	Needles	Taxol: 0.00183%–0.00406% dw	[55]
			10 DAB III: 0.02476%–0.05949% dw	
	Netherlands and/or UK		Taxol: 0.0272% dw	[53]
			Cephalomannine: 0	
			Baccatin III: 0	
			10 DAB III: 0.1092% dw	
*T. yunnanensis*	China	Stem bark	Taxol: 0.024%–0.030% dw	[49]
			Cephalomannine: 0.0088%–0.018% dw	

10 DAB III: 10 deacetyl baccatin III; fw: fresh weight; dw: dry weight; n.d.: nondetectable; n.q.: not quantifiable;

* plant age = 15 years;

** obtained by using SPE-HPLC;

*** obtained by using TLC-HPLC;

**** plant age from 27 to 136 years and the concentration was expressed by gram per each plant;

***** obtained from a variety of sources/multiple times;

Z,W,K and C samples collected from Zielona Gora, Warsaw, Koszalin, and Cracow sites, Poland, respectively.

**Table 2:** Constituents of oils of different *Taxus* species worldwide.

*Taxus* species	Plant part	The most dominant components (%)	Extraction Method	Country	Analysis method	References
*T. baccata*	Fresh leaves	^A^1-Octen-3-ol (32.4%); *trans*-2-hexen-1-ol (8.2%); caryophyllene oxide (7.2%) and hexahydrofarnesyl acetone (6.8%)	Hydrodistillation	Turkey	GC and GC-MS	[4]
		^M^1-Octen-3-ol (20.7%); 1-hexanol (10.9%) and *trans*-2-hexen-1-ol (7.3%)				
	Fresh needles and twigs	a1-Octen-3-ol (15.56%); myrtenol (13.30%) and *cis*-3-hexen-1-ol (6.84%)	Hydrodistllation in a Clevenger-type apparatus	Serbia	GC-FID and GC-MS	[5]
		b1-Octen-3-ol (27.55%); myrtenol (12.88%) and *cis*-3-hexen-1-ol (4.77%)				
		c1-Octen-3-ol (22.18%); *cis*-3-hexen-1-ol (19.78%) and myrtenol (9.22%)				
		d1-Octen-3-ol (23.48%); *cis*-3-hexen-1-ol (11.46%) and myrtenol (11.38%)				
	Fresh needles and branches	Hexahydrofarnesyl acetone (18.3%); myrtenol (18.3%); *cis*-3-hexen-1-ol (6.0%); senecioic acid (5.9%) and tricosane (5.5%)	Hydrodistllation in a Clevenger-type apparatus	Serbia	GC and GC-MS	[80]
	-	1-Octen-3-ol (>50%), eugenol (0.5–5%) and *cis*-3-hexen-1-ol (<0.5%)	Hydrodistillation followed by enzymatic hydrolysis with -glucosidase	Netherlands	GC and GC-MS	[1]
*T. baccata L*.	Leaves	Oleic acid (20.87 %); 9,12-octadecadien-1-ol (17.77 %); 4-hydroxyphenylacetic acid (9.67 %); 2-methyl-1-thia-cyclopentane (8.87%); 3,5-dimethoxyphenol (7.65%) and pluchidiol (5.05%)	Water:methanol extract	Iran	GC-MS	[11]
	Male Cones	3-O-methyl-D-glucose (64.00%); oleic acid (13.32%); 9,12-octadecadien-1-ol (7.70%) and 2-ethylidene-6-methyl-3,5-heptadienal (2.66%)				
	Fresh needles	Palmitic acid (19.6%); capric acid (19.5%); lauric acid (8.1%); decanol (5.4%) and ethyl linolenate (4.2%)	Enzymatic Hydrolysis followed by hydrodistllation in a Clevenger-type apparatus	Turkey	GC-MS	[10]
	Dried needles	Palmitic acid (22.5%); capric acid (12.6%); myristic acid (8.0%); lauric acid (5.9%) and hexahydrofarnesyl acetone (4.7%)				
	Red arils	^Z^Linoleic acid (30.92%); palmitic acid (20.43%); α-linolenic acid (18.53%); myristic acid (9.84%) and oleic acid (9.52%)	Folch’s method with chloroform-methanol mixture (2:1, v/v)	Poland	GC-FID	[9]
		^W^α-Linolenic acid (25.18%); palmitic acid (22.66%); linoleic acid (20.99%); myristic acid (10.76%) and oleic acid (6.65%)				
		^K^α-Linolenic acid (23.43%); palmitic acid (22.37%); linoleic acid (21.33%); oleic acid (12.35%) and myristic acid (6.76%)				
		^C^α-Linolenic acid (26.50%); palmitic acid (24.37%); linoleic acid (19.40%); myristic acid (10.39%) and oleic acid (6.59%)				
	Seeds	Oleic acid (54.78%); linoleic acid (23.08%) and taxoleic acid (9.50%)	Folch’s method with chloroform-methanol mixture (2:1, v/v)	Britain or France	GLC	[15]
	Seeds	Oleic acid (59.3%); linoleic acid (16.8%) and taxoleic acid (12.2%)	Petroleum ether extract	USA	GLC	[12]
	Seeds	Oleic acid (56.00%); linoleic acid (22.81%) and taxoleic acid (9.57%)	Folch’s method with chloroform-methanol mixture (2:1, v/v)	France	GLC	[14]
*T. canadensis*	Fresh twigs and needles	1-Octen-3-ol (44.64%) and *trans*-2-hexenal (24.13%)	Steam distillation	Canada	GC-MS	[2]
		3,5-Dimethoxyphenol (48.65%); 1-octen-3-ol (23.05%) and *cis*-3-hexen-1-ol (3.68%)	Enzymatic hydrolysis with -glucosidase			
		1-Octen-3-ol (39.11%); 3,5-dimethoxyphenol (26.29%) and *cis*-3-hexen-1-ol (4.09%)	Enzymatic hydrolysis with cellulose			
	Seeds	Oleic acid (46.77%); linoleic acid (27.93%) and taxoleic acid (13.65%)	Bligh and Dyer method using chloroform and methanol	Canada	GLC-FID	[13]
	Leaves	1-Propanone (36.38%); morpholine (10.95%); methylamine (9.10%); methanone (8.14%) and caryophylleneoxide (4.05%)	HS-SPME	Canada	GC-MS	[75]
*T. chinensis*	Stems	α-Pinene (34.8%); caryophyllene oxide (17.1%); *trans*-verbenol (5.0%) and verbenone (4.6%)	Hydrodistllation in a Clevenger-type apparatus	Vietnam	GC-FID and GC-MS	[7]
	Leaves	α-Pinene (24.2%); sabinene (19.5%); α-terpinyl acetate (12.8%); 1,8-cineole (11.7%); β-pinene (6.1%) and manoyl oxide (4.3%)	Hydrodistllation in a Clevenger-type apparatus	Vietnam	GC and GC-MS	[8]
	Woods	α-Pinene (20.0%); photosantalol (10.2%); caryophyllene oxide (8.9%); spathulenol (7.6%); guaiol (6.8%); β-pinene (5.6%) and bornyl acetate (5.4%)				
	Seeds	Oleic acid (34.31%); linoleic acid (34.22%) and taxoleic acid (16.08%)	Folch’s method with chloroform-methanol mixture (2:1, v/v)	Britain or France	GLC	[15]
	Bark	Elemicin (47.50%); 4,6-diamino-3-[4-methoxyben zyl]-1H-pyrazolo[3,4-d]pyrimidine (3.21%) and butyl isodecyl phthalate (0.63%)	Ethanol extract	China	GC-MS	[6]
		Elemicin (29.89%) and asarone (0.53%)	Ethanol/methanol mixture extract			
		Elemicin (46.23%); diisobutyl phthalate (3.11%); 4,6-diamino-3-[4-methoxybenzyl]-1H-pyrazolo[3,4-d]pyrimidine (3.11%) and dibutyl phthalate (2.32%)	Ethanol/benzene mixture extract			
	Sapwood	Elemicin (30.61%) and γ-sitosterol (2.29%)	Ethanol extract			
		Elemicin (18.24%); 2,3,5,6-tetrahydro-3,3,4,5,5,8-hexamethyl-s-indacene-1,7-dione (14.46%); macckiain (5.12%) and 4,6-diamino-3-[4-methoxybenzyl]-1H-pyrazolo[3,4-d]pyrimidine (2.57%)	Ethanol/methanol mixture extract			
		Elemicin (29.69%); laminitol (5.16%); γ-sitosterol (2.52%) and diisobutyl phthalate (2.13%)	Ethanol/benzene mixture extract			
	Heartwood	Formononetin (17.71%); laminitol (8.19%); pseudobaptigenin (5.40%); 2,3,5,6-tetrahydro-3,3,4,5,5,8-hexamethyl-s-indacene-1,7-dione and macckiain (2.32%)	Ethanol extract			
		Elemicin (4.69%); laminitol (3.79%) and nerolidol (1.27%)	Ethanol/methanol mixture extract			
		Laminitol (14.48%); nerolidol (7.04%); γ-sitosterol (4.99%); diisobutyl phthalate (3.82%); 3-O-methyl-D-glucose (3.33%) and dibutyl phthalate (2.76%)	Ethanol/benzene mixture extract			
*T. chinensis* var. *mairei*	Leaves	*cis*-Vaccenic acid (36.96%); *trans*-palmitoleic acid (24.05%); palmitic acid (6.19%); hexadecanoic acid methyl ester (4.82%) and ethyl oleate (3.37%)	Hydrodistllation in a Clevenger-type apparatus	China	GC-MS	[77]
		*cis*-Vaccenic acid (36.73%); *trans*-palmitoleic acid (23.66%); palmitic acid (6.19%); hexadecanoic acid methyl ester (4.84%) and ethyl oleate (3.44%)	Microwave-assisted simultaneous distillation extraction			
	Aerial stems	^H^Phthalic acid mono-2-ethylhexyl ester (21.36%); palmitic acid (16.60%); butylated hydroxytoluene (7.75%); stearic acid (7.27%) and ethylbenzene (5.04%)	SFE-CO2 extraction	China	GC-MS	[78]
		^Q^Phthalic acid mono-2-ethylhexyl ester (25.21%); palmitic acid (19.37%); 7,9-di-tert-butyl-1-oxaspiro (4,5) deca-6,9-diene-2,8-dione (9.69%); ethylbenzene (6.36%); stearic acid (6.29%) and butylated hydroxytoluene (5.71%)				
		^S^Phthalic acid mono-2-ethylhexyl ester (26.38%); palmitic acid (12.31%); butylated hydroxytoluene (7.51%) and stearic acid (5.06%)				
		^X^Heptacosane (24.93%); palmitic acid (5.97%) and 7,9-di-tert-butyl-1-oxaspiro (4,5) deca-6,9-diene-2,8-dione (5.82%)				
	Leaves	Benzene propanenitrile (49.39%); 1-hydroxy-2-butanone (12.72%); acetic acid (5.39%); 1-octen-3-ol (4.28%) and *trans*-2-hydroxycinnamic acid (3.53%)	Steam distillation	China	GC-MS	[73]
*T. cuspidata*	Fresh stems	Ethyl linoleolate (9.0%); longiborneol (7.9%); 13-diepoxy-14,15-bisnorlabdane (7.0%) and ambrettolide (4.5%)	Microwave-assisted hydrodistillation	Korea	GC-MS	[76]
	Seeds	Oleic acid (39.21%); linoleic acid (29.35%) and taxoleic acid (16.16%)	Folch’s method with chloroform-methanol mixture (2:1, v/v)	Britain or France	GLC	[15]
	Seeds	Oleic acid (36.50%); linoleic acid (32.88%) and taxoleic acid (16.02%)	Bligh and Dyer method using chloroform and methanol	Japan	GLC-FID	[13]
	Leaves	Ethyl phthalate (28.15%); *E*-procainamide (4.59%); 3-methyl-4,4-diphenyl-2-cyclohexen-1-one (4.20%) and n-hexyl vinyl alcohol (3.54%)	Microwave-assisted hydrodistillation	Korea	GC-MS	[74]
*T. media*	Leaves	Benzene propanenitrile (21.30%); 1,4-dioxane-2,3-diol (20.13%); 3-bromo-3-methyl-butyric acid (17.92%) and 1-hydroxy-2-butanone (9.85%)	Steam distillation	China	GC-MS	[73]
*T. wallichiana*	Fresh leaves	*trans*-2-Octen-1-ol (14.5%); pentacosane (8.1%); caryophyllene oxide (7.1%); 1-octanol (6.5%); caproic acid (5.5%) and *cis*-3-hexen-1-ol (4.1%)	Hydrodistllation in a Clevenger-type apparatus	India	GC-MS	[72]
*T. wallichiana* var *mairei*	Leaves	*cis*-3-Hexen-1-ol (12.14%); 1-octen-3-ol (9.56%); 2-hexenal (7.45%); hexyl formate (4.24%); 2-penten-1-ol (3.71%); 3-octanone (3. 65%) and 1-penten-3-ol (3.51%)	Simultaneous distillation and diethyl ether extraction	China	GC-MS	[3]
		2-Hexenal (7.03%); *cis*-3-hexen-1-ol (4.99%); palmitic acid (4.77%); hexanol (4.44%) and 3-octanone (4.06%)	Simultaneous distillation and dichloromethane extraction			

a Population I/Tara,

b Population II/Kopaonik,

c Population III/Malinik and

d Population I – III, Serbia.

Z,W,K and C Samples from Zielona Gora, Warsaw, Koszalin and Cracow sites, Poland, respectively.

H,Q,S and X Plant samples collected respectively from Huangshan city, Qingyang county, Shucheng county and Xuancheng city, China. GLC: Gas–liquid chromatography. GC-MS: Gas chromatography–mass spectrometry. SFE-CO_2_: Supercritical fluid extraction using carbon dioxide.

A and M Samples collected from western (Aegean region) and southern (Mediterranean region), Turkey, respectively. HS-SPME: Head space solid phase micro-extraction. − : Missing data.

**Table 3:** Antimicrobial activities of the oils of the species of the genus *Taxus* worldwide.

*Taxus* species	Plant part	Sample	ZI (mm)	MIC value	MBC value	IC_50_	Bacterial strain	References
*T. chinensis*	Leaves	Essential oil extracted by hydrodistllation in a Clevenger-type apparatus	-	16.0 μg/mL	-	3.98 μg/mL	*E. faecalis* ATCC 299212	[8]
			-	256.0 μg/mL	-	100.56 μg/mL	*S. aureus* ATCC 25923	
			-	64.0 μg/mL	-	19.78 μg/mL	*B. cereus* ATCC 14579	
			-	NA	-	NA	*E. coli* ATCC 25922	
			-	NA	-	NA	*P. aeruginosa* ATCC 27853	
			-	NA	-	NA	*S. enterica* ATCC 13076	
			-	128.0 μg/mL	-	55.67 μg/mL	*C. albicans* ATCC 10231	
	Woods		-	64.0 μg/mL	-	20.33 μg/mL	*E. faecalis* ATCC 299212	
			-	128.0 μg/mL	-	56.78 μg/mL	*S. aureus* ATCC 25923	
			-	NA	-	NA	*B. cereus* ATCC 14579	
			-	256.0 μg/mL	-	87.78 μg/mL	*E. coli* ATCC 25922	
			-	NA	-	NA	*P. aeruginosa* ATCC 27853	
			-	NA	-	NA	*S. enterica* ATCC 13076	
			-	256.0 μg/mL	-	89.67 μg/mL	*C. albicans* ATCC 10231	
*T. chinensis* var. *mairei*	Leaves	Essential oil obtained by steam distillation	-	95%	-	-	*E. coli*	[73]
			-	98%	-	-	*S. aureus*	
*T. cuspidata*	Leaves	Essential oil isolated by microwave-assisted hydrodistillation	34.0	250 μg/mL	500 μg/mL	-	*B. cereus* ATCC 13061	[74]
			27.0	500 μg/mL	1000 μg/mL	-	*L. monocytogenes* ATCC 7644	
			34.0	250 μg/mL	500 μg/mL	-	*S. aureus* ATCC 12600	
			22.0	500 μg/mL	1000 μg/mL	-	*S. typhimurium* ATCC 43174	
			24.0	500 μg/mL	1000 μg/mL	-	*E. coli* ATCC 43889	
*T. media*	Leaves	Essential oil extracted by steam distillation	-	5%	-	-	*E. coli*	[73]
			-	5%	-	-	*S. aureus*	

ZI: Zone of inhibition. MIC: Minimum inhibitory concentration. MBC: Minimum bactericidal concentration.

**Table 4:** Antioxidant activities of the oils of *Taxus* species.

*Taxus* species	Plant part	Sample	Assay	Inhibitory effect (%)	References
*T. cuspidata*	Fresh stems	Essential oil extracted by microwave-assisted hydrodistillation	DPPH	92.8%^a^	[76]
			Nitric oxide radical	80.0%^b^	
			Superoxide radical	71.7%^c^	
			Hydroxyl radical	73.7%^d^	
			Lipid peroxidation	80.2%^e^	
			Reducing power activity	1.1^f^	

a At the concentration of 500 *μg*/mL.

b At the concentration of 300 *μg*/mL.

c At the concentration of 250 *μg*/mL.

d At the concentration of 500 *μg*/mL.

e At the concentration of 250 *μg*/mL.

f Absorbance value at 25 *μg*/mL concentration.
